# Exploring infant feeding practices and associated factors among HIV-positive mothers attending early infant diagnosis clinic in Northern Uganda

**DOI:** 10.1017/S0950268822001091

**Published:** 2022-06-20

**Authors:** Ivan Mutawulira, Jane Nakachwa, Laymond Muharabu, Abel Wilson Walekhwa, Vincent Kayina

**Affiliations:** 1 School of Medicine, Gulu University, P.O.Box 166, Gulu, Uganda; 2 School of Public Health, Makerere University P.O.Box 7072, Kampala, Uganda; 3 Makerere University College of Veterinary Medicine, Animal Resources and Biosecurity, P.O.BOX 7062, Kampala, Uganda

**Keywords:** Exclusive breastfeeding, Gulu, HIV positive, infant feeding, Uganda

## Abstract

This study assessed the infant feeding practices and their determinants among human immune deficiency virus (HIV)-positive mothers with infants (0–12 months).

A cross-sectional study design adopting qualitative and quantitative data collection procedures was used. This study was carried out from Awach Health Center IV in Gulu city, Uganda. We enrolled 108 adult participants who were HIV-positive mothers with an infant from October to December 2021. Semi-structured questionnaire and focused group discussion (FGD) guide were used in data collection. Data were collected, edited, coded and entered into Epi info. The data were analysed using SPSS version 22. Qualitative data were analysed using Atlas.ti software.

Of the 108 mothers, 83/108 (77%) practised exclusive breastfeeding (EBF) while 25/108 (23%) practised mixed feeding. Qualitative results also showed that EBF was the preferred choice. Majority of the respondents 104/108 (96.3%) believed HIV can be transmitted to their babies. Factors associated with infant feeding practices at multivariant level analysis at 5% significance were age of the child (OR 0.706, 95% CI 0.210–0.988), income level (OR 1.296, 95% CI 1.150–10.631).

Majority of mothers had good knowledge about the prevention of mother-to-child transmission. Mothers should deliver from hospitals, more sensitisations for these mothers to appreciate the benefits of EBF.

## Background

Exclusive breastfeeding (EBF) is defined as feeding infants only on breast milk, be it directly from the breast or expressed, except drops or syrups consisting of vitamins, mineral supplements or medicine [1]. The World Health Organization (WHO) guidelines recommend that in settings where breastfeeding is judged to be the safest infant feeding option, mothers with human immune deficiency virus (HIV) should exclusively breastfeed their infants for the first six months and continue to breast feed for at least 12 months while introducing complementary foods [2]. Even with these guidelines, mothers do not practice it because of a range of reasons [3]. Thereby creating a cave of early infant diagnosis (EID) of morbidities to infants as it is influenced by the feeding choice that the mothers make [4].

Although there is a risk of HIV infection through breastfeeding, the risk of mortality and morbidity is higher if infants are taking replacement feeding [5]. In many resource-limited settings, HIV-exposed infants (HEI) who are not breastfed were up to six times more likely to die from diarrhoeal diseases, malnutrition and pneumonia [6]. The benefits of breastfeeding are incomparable and comprise the prevention of infant infectious diseases and reduction of leukaemia, sudden infant death syndrome, type 1 and 2 diabetes and obesity [7]. EBF for the first 6 months of the infants carries a 4–10-fold decreased risk of MTCT of HIV compared to mixed breastfeeding [8].

Breast feeding is an important component of the wellbeing and survival of children breast milk provides optimal nutrition, contains anti-bodies that protect infants and is unlikely to become contaminated [9]. The overall prevalence of EBF in sub-Saharan Africa is low which was at 36%, prevalence was the highest in Rwanda and the lowest in Gabon, despite the fact that Sub-Saharan Africa is the home of higher rates of MTCT, malnutrition, infant and mortality rate [10].

The current evidence suggests that high-income countries practice a shorter duration of breastfeeding compared with low- and middle-income countries. However, within low- and middle-income countries, approximately only 37% of infants younger than six months are exclusively breastfed. Just scaling up and promoting breastfeeding to the universal level could prevent 823 000 annual death in children under 5 years and 13 810 of these under 2 years [11]. In Uganda a study done showed that the proportion of HEI that were exclusively breastfed reduced with increasing age of the infant, the incidence of non-exclusively breastfeeding at 6 weeks and 14 weeks post-partum were 22.5% and 42.9%. By the time infants were 14 weeks of age almost half of them were not exclusively breastfeeding [12]. Several studies have shown low rates of EBF in outside countries however very few of such studies have been done in Uganda particularly Northern region. Therefore, the aim of this study was to assess the infant feeding practices among HIV-positive mothers and the factors that influence the choice of the feeding practices.

## Methods

### Study area

**Fig. 1.**
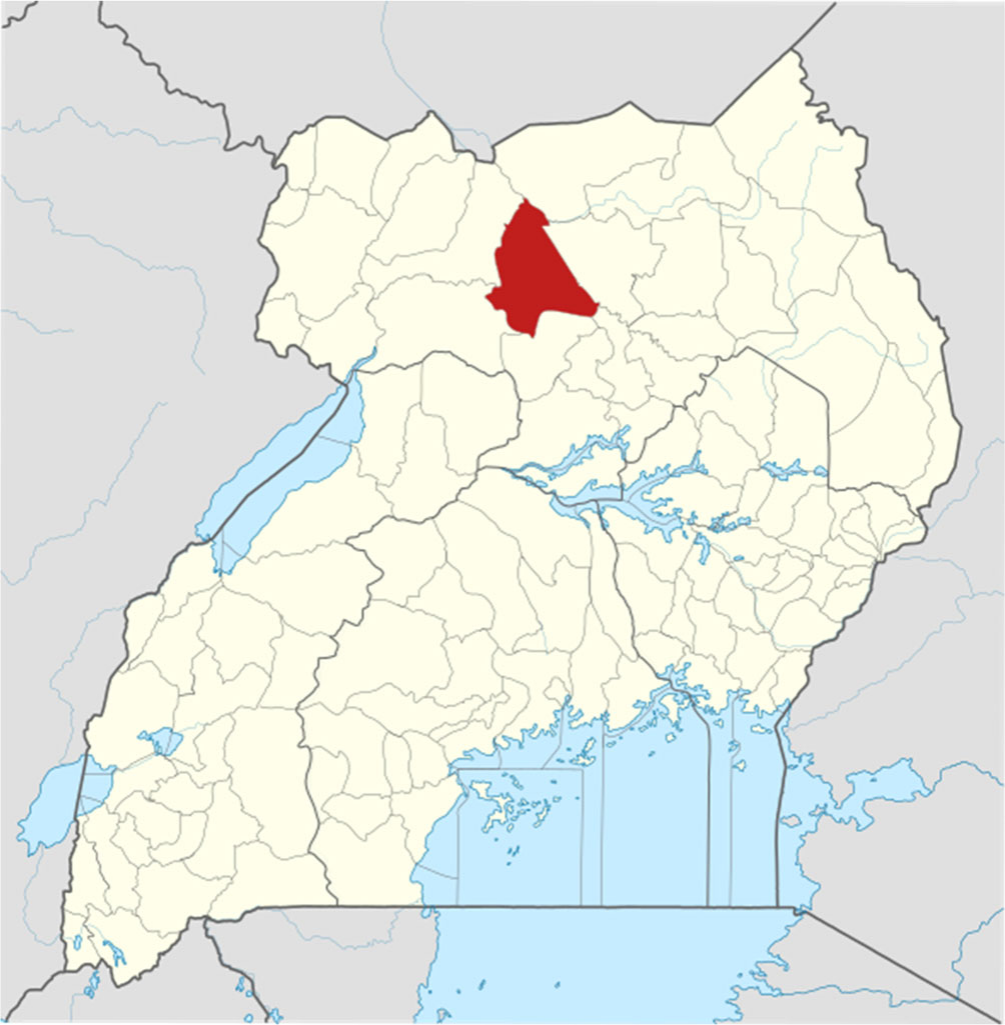
Map of Uganda showing location of Gulu District.

The study was conducted from Awach Health Centre IV in the HIV EID clinic. EID is a specialised clinic where HEI and their mothers seek care. Awach Health Centre IV is found in Awach subcounty, which is found in Aswa County in Gulu district, Northern Uganda. It is about 35.1 km from Gulu city via Gulu-Patiko road, and Gulu city is 335 km from Kampala the capital city of Uganda, it is located on latitude 3 001 011and longitude 3 202 315 911 (Fig. 1, this map was adapted from [13]). This is the major health centre in Aswa health sub district, it is located north of Paroma close to Awach public nursery and primary school. Awach subcounty has 4 parishes and 32 villages. Has a population of 22 576. Major economic activities are trade through business and subsistence farming. The quickest transport in the area is by use of commercial motorcycle. Major language spoken in Gulu is Acholi.

### Study population

The study population consisted of all mothers who were HIV positive and having a child (0–12) months from 16–43 years, attending EID from Awach Health Centre IV and were present during the time of data collection. These data were collected every Thursday where about 25–30 HIV-positive breastfeeding mothers came to the facility to get their medications.

### Sample size

The Kish and Leslie formula of sample size determination was used to generate the minimum number of HIV-positive mothers required for the study [14], giving us a sample size of 108 participants.

### Sampling procedure

We used simple random sampling during each day of quantitative data and purposive sampling for qualitative data collection. During simple random sampling, we used small pieces of paper which we labelled ‘yes’ and ‘no’, then they were put in a container and eligible participants were asked to pick a paper among those labelled. Those who picked ‘yes’ consented and were interviewed. For example, if on a given day, we had averagely 30 participants and we interviewed all the eligible and consenting participants, therefore we made 30 pieces had ‘yes’ and the rest pieces had ‘no’, then the pieces were distributed to all the participants those who chose yes were interviewed. Only those who consent to participate in the study would be subjected to picking of papers. We held two focussed group discussion (FGD) where participants were selected purposively. We picked 5 Key informants because they had knowledge about breast feeding practices in the HIV/Acquired Immunodeficiency Syndrome (AIDS) context, these were, 2 PMTCT nurses, 2 midwives and I councillor.

### Data collection tools

Quantitative data were collected by trained and experienced research assistants using a brief interviewer administered questionnaire. Qualitative data were collected using FGD guide and key informant guide. The collection tools were pretested from Atanga health centre III which neighbours the study site.

### Data collection procedure

The data were collected for over a period of 6 weeks. With the help of the nurses, the eligible participants were chosen then an interviewer semi-structured questionnaire was used on respondents who were randomly selected and consented. The questions were centred on infant feeding practices and associated factors. The data were collected by the research team. The variables of interest included; the socio-demographics of the mothers, their knowledge and understanding of infant feeding, observed practices during infant feeding and the attitude about infant feeding.

### Data management and analysis

The quantitative data were analysed using a statistical package for the social sciences (SPSS) for windows version 22. Furthermore, qualitative data were analysed by deductive thematic analysis using ATLAS.ti software. Descriptive statistics including measures of central tendency (that is mean and median) and dispersion as standard deviation (s.d.) and range, as well as frequencies and percentages were used to describe the study population. The infant feeding practices of the mothers were described using pie charts and bar graphs. Bivariate analysis (odds ratio) was conducted to establish associations between various variables and infant feeding practices. Variables which showed significant association were further subjected to multivariate analysis (logistic regression) to establish the determinants of infant feeding practices. A *P*-value of <0.005 was used as the basis for statistical significance.

Qualitative interviews were conducted in English using KI and FGD guides. Each interview was transcribed real time by the research assistant. A total of 57 codes for KIs and 11 for FGD were originally generated. The codes generated were analysed for consistence and either divergence or convergence. Those that converged formed a particular theme and this was deduced by the data analysis team. Those that diverged also formed a particular theme and this was also noted. The codes were developed from the objective of the study and transcribed data, and then entered into ATLAS.ti version 8 software for analysis. Using deductive thematic analysis, the categorised data were used to develop main themes which made the final results of our study. A total of 4 main themes emerged from this data analysis, the included (1) barriers to elimination of mother-to-child transmission (EMTCT), (2) common infant feeding practices among the mothers, (3) roles of health workers on the choice of infant feeding practice among HIV-positive mothers, (4) factors that influence the choice of the infant feeding practice. These themes were documented as key outcomes of our study and consequent quotes attached quotes attached to back up our findings. For the quality control and quality assurance of the transcripts, two FGD transcripts were randomly selected to check for accuracy by a senior member of this team. He listened to the recording and compared with the transcripts that had been transcribed.

### Ethical considerations

Ethical approval for the study was obtained from Gulu University Research Ethics Committee on the 14 October 2021 IRB No. GUREC-2021-76 (supplementary 1). Permission was sought from the administration of Awach Health Centre IV and obtained on the 15 October 2021. Participants were fully informed about the study and written consent was sought from all participants before anyone was interviewed. Any participant was free to accept or decline in participating in the study. Confidentiality was guaranteed by ensuring that the participant's names are not known but questionnaires were just numbered. The participants had a right to withdraw from the interview at any given time of the interview. The participants were allowed to ask questions before, during and after the study, any time of their convenience. The participants were informed that after the study they will not receive any incentive.

The researcher administered the questionnaire in a privately secluded room to ensure the privacy and confidentiality of these respondents

## Results

- Quantitative results

### Socio-demographic and socio-economic characteristics of participants

Response, a total of 108 HIV-positive mothers were surveyed with 100% response rate. A total of 108 mothers participated in the study, making the response rate of 100%. The age range of the study population was from 16 to 43 years. The mean age of the mothers was 28 + 6.07 years while the mean age of the infants was 5.7 + 2.86 months. Majority 89 (82.4%) of the participants were unemployed, 91 (84.3%) had an income of one hundred thousand per month and majority (71%) had no house helpers (Table 1).

**Table 1.** Sociodemographic characteristics of the participants (9–13 years)

Characteristics	Measures	Frequency (*N* = 108)	Percentage (%)
Age of the child (months) Mean + s.d.	5.7 + 2.86		
0–6 months		68	63.0
7–12 months		40	37.0
Mothers age (years) Mean + s.d.	28 + 6.07		
16–25		40	37.0
26–35		55	50.9
36–45		13	12.0
Employment status			
Salaried job		7	6.5
Self employed		12	11.1
Unemployed		89	82.4
Average monthly income (USD)			
29		91	84.3
29–58		7	6.5
>59		10	9.3
House helper for house work?			
Yes		31	28.7
No		77	71.3

### Infant feeding practices

#### Proportion of HIV-positive mothers practising the different infant feeding practices

**Fig. 2.**
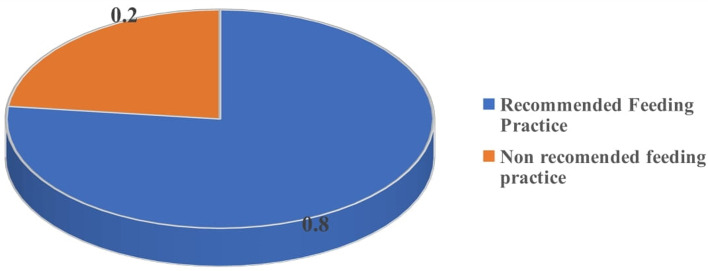
Proportion of HIV/AIDS Positive mothers practising different infant feeding practices.

Out of the 108 HIV-positive mothers studied, 77% of the mothers practised recommended feeding practice for the first 6 months (Exclusive breast feeding) while 23% of the mothers practised mixed infant feeding practices (Fig. 2).

#### Knowledge of HIV-positive mothers on EMTCT in Awach H/C IV

The results in Table 2 below summarises the knowledge of mothers on EMTCT. On the transmission of HIV, mothers had high knowledge 104 (96.3%) of the mothers believe HIV-positive mothers can transmit HIV virus to the babies. Out of the total respondents, mothers had medium knowledge 61 (56.5%), and low knowledge 40 (37%), knew that MTCT of HIV virus can occur breast feeding and during delivery respectively. 97.2% and 96.3% of the mothers believe MTCT of HIV can be prevented and EMTCT is important for both the child and the mother. Almost average (44.4%) of the mothers had attended EID clinic at least three times.

**Table 2.** Knowledge of HIV-positive mothers on EMTCT in Awach H/C IV

Characteristics	Frequency (*N* = 108)	Percentage (%)
Can HIV-positive mothers transmit HIV to babies		
Yes	104	96.3
No	3	2.8
I don't know	1	0.9
When can HIV be transmitted from mother to child		
During pregnancy	6	5.6
During delivery	40	37.0
During breast feeding	61	56.5
I don't know	1	0.9
Is MTCT of HIV preventable		
Yes	105	97.2
No	3	2.8
Is EMTCT important for child/mother		
Yes	104	96.3
I don't know	4	3.7
How many times have you attended EID clinic?		
1–3 times	48	44.4
4–6 times	34	31.5
More than 6 times	26	24.1

#### Factors associated with infant feeding methods

1. Qualitative results

## Barriers to EMTCT

- Long distance to the facility

The distance to the facility poses a big challenge to EMTCT. The long distance makes majority of the mothers fail to follow their scheduled dates hence leading to poor adherence (Table 3)

*‘Long distance, delays or leads to failure to turn up sometimes hence makes mothers not to comply to their treatment’* ***K1 (Male Health worker)***

- Inadequate human resource

The number of health workers in the facility affects effective programme of EMTCT. Due to the few numbers, less time is given to the mothers. The low number coupled with higher number of patients also leads to long waiting some of them go away before being attended to especially counselling and attending to their issues.

*‘Human resource is a major problem, because when we take this unit, we are only three and at times you need to go out and follow up so it is not easy’ **K2 (Female Health worker)***

**Table 3.** Multivariable analysis of factors associated with the choice of infant feeding practices among HIV-positive mothers

Characteristics	AOR	(95% CI)	*P*-Value
Age of the child			
0–6 months	1.0		
7–12 months	0.706	(0.210–0.988)	0.043*
Average monthly income			
29	1.0		
>59	1.296	(1.150–10.631)	0.013*
Is MTCT of HIV preventable			
Yes	1.0		
No	16	(3.618–34.18)	0.040
Number of EID clinic attendance?			
1–3 times	1.0		
4–6 times	0.303	(0.090–1.000)	0.05*
More than 6 times	0.118	(0.019–0.704)	0.019*
Where did you deliver from?			
Home	1.0		
Facility	1.976	(1.295–13.233)	0.038*

### Common infant feeding practices among the mothers

The most common infant feeding practice as indicated by both the KI and FGD was EBF for six months. This the recommended infant feeding practice among HIV-positive mothers.

*‘exclusive breastfeeding for six months if the mother has no problem with the breast, this is what most mothers do for the first six months since they cannot afford exclusive formula feed’* ***K5 (Female Councillor)***

### Roles of health workers on the choice of infant feeding practice among HIV positive mother

According to the key informants, health workers have a big role to play in influencing mothers in the choice of the infant feeding practice.

*‘yes, we encourage exclusive breastfeeding because it is safe in preventing infections to the mothers and is cheap for the mothers to practice’ **(K3 (Female Health worker)-***

*‘Yes, we provide the mothers with information about the different infant feeding practices including the benefits and disadvantages of each method, we even counsel them which one to use’ **K4 (Male Health worker)***

### Factors that influence the choice of infant feeding practices

According to the findings from the interview and the FGD's. there were many factors elaborated to have influence on the mother's choice of the feeding practice. These factors included poverty of lack of resources for exclusive formula feeding (EFF), social influence on the mothers, lack of adequate information.

*‘poverty, I would prefer my child to feed on formula milk so that I can reduce the risk of transmission but I don't have the money to buy. **K5 (Female Councillor)***

*‘I also think some mothers do not have enough information about the different feeding options**’ K5 (Female Councillor)***

*‘economic status, mothers would want to choose a safer method for example formula feeding but it is expensive’* ***K2 (Female Health worker)***

## Discussion

In this study, we assessed the infant feeding practices and associated factors among HIV-positive mothers. We found out that 83/108 (77%) practice EBF among those below six months, 25/108 (23%) practice mixed feeding. On transmission of HIV/AIDS, 104/108 (96.6%) believe HIV can be transmitted from the mother to the babies. Out of the total respondents 61/108 (56.4%) and 40/108 (37%) believe HIV can be transmitted during breastfeeding and delivery respectively. Factors associated with the infant feeding practice included, age of infant, EID visits, place of delivery, income level. Qualitative analysis showed that the most practised infant feeding was EBF, barriers to EMTCT included long distance and inadequate staff, factors influencing the choice of infant feeding practice included, poverty, social influence on the mother, influence of the health workers.

The findings of this study revealed that majority 83/108 (77%), practised EBF. This could be attributed to the good PMTCT counselling services, the improvement of Antenatal Care (ANC), and bringing such services closer to the communities. This finding was in line with various studies done from Ethiopia and Kenya that also showed that majority of the mothers practised EBF. However, the findings of this study were lower than those found in a study done in Ethiopia that showed 89% practising EBF [15]. Also, lower than a study done in Debre Markos Referral hospital that showed that 85.8% practised EBF [16]. This could be attributed to the fact that these studies were done in an Urban setting, and probably these respondents were more informed about the benefits of breast milk in an HIV/AIDS context. Also, these urban people could be exposed to literature through TVs and radios about the benefits of breastmilk. Their educational status was also higher than the one in our study. On the contrary, the findings of this study were higher than other studies for example a study done in Kenya showed that 71.4% [17], slightly lower than ours. This can be explained that in the study a certain percentage could have practised formula feeding. Our study further noted that 25/108 (23%), close to a quarter of the mothers practised mixed infant feeding. This finding was higher than a study than in Kenya that showed that 18.2% practised mixed feeding [17]. The rate of mixed feeding in this study was also higher than in the study done in Ethiopia (14.2) [16]. None of the participants practised formula feeding. There was no formula feeding most likely because it was not readily accessible, feasible and affordable in a rural area like Awach.

On transmission of HIV, majority, 104 (96.3%) of the mothers believed HIV-positive mothers can transmit HIV to the babies. This awareness can be attributed to the increased attendance of ANC, and creating an EID clinic so that the mothers can continuously be sensitised. This is in agreement with a study done in Lesotho where 96% believed HIV can be transmitted to their babies [15]. However, another study done in Malawi showed very low comprehensive knowledge about PMTCT (52%) [18] this could be because of low PMTCT services in the country. Out of the total respondents, 61/108 (56.5%), and 40/108 (37%), knew that MTCT of HIV virus can occur during breast feeding and delivery respectively. These findings were lower than findings in a research held in Ethiopia that showed that 84% and 87.8% knew that HIV can be transmitted during delivery and breastfeeding respectively [19]. 97.2% and 96.3% of the mothers believed MTCT of HIV can be prevented and EMTCT is important for both the child and the mother. This could be explained by bringing ANC closer to the population that is to say Health Center IIs and village levels, and probably the health workers being closer to the mothers and teaching them about PMTCT in a friendly way. Almost average (44.4%) of the mothers had attended EID clinic at least three times. This knowledge coupled with boosted EID clinic attendance can help to eliminate HIV transmission to the infants because mothers are more likely to be cautious about preventive measures taught by health workers.

Exclusively breastfed infants reduced with the age of the child (OR 0.706, 95%). This could be explained by the fact that in the early months of life, mothers give more attention to their children and stay with them longer hence most likely to exclusively breastfeed. Compared to later months of life where mothers perceive that the child has grown and the breast milk may not be enough hence propelling the mother to start mixed feeding. This is in line with a study done in Lira Uganda that showed HEI that were exclusively breasted reduced with increasing age [12]. We also found out that Older mothers were 1.545 times more likely to practice EBF compared to young mothers. This could be explained by the fact the older mothers were more likely to have other children and so could have been counselled and sensitised about the benefits of breastfeeding compared to their counterparts. This agrees with a study done in Lesotho that revealed that mothers who had earlier feeding counselling were more likely to practice EBF compared to late counselling [15]. The study further revealed that Educated mothers were 4.3 times more likely to practice recommended infant feeding practices compared to uneducated. This could be explained by the fact that educated mothers are more likely to comprehend the benefits of EBF, and also have high chances of getting exposed to good information about recommended infant feeding practices through TVs and radios. This was in line with a study done in Kenya where education had an OR 0.27 on determinants of complimentary feeding [17]. Our study found out that Mothers who delivered from a health facility were 1.97 times more likely to follow recommended infant feeding practices compared to those who delivered from home. This may be due to the counselling these mothers get from the midwives during delivery and encouragement from their fellow mothers. This is comparable to a study done in rural South Nyanza Kenya which revealed that hospital delivery was associated with high rates of EBF [20].

According to the findings from the interviews and FGDs, there were many factors elaborated to have influence on the mother's choice of the feeding practice. These factors included, poverty with lack of resources for EFF, social influence on the mothers, lack of adequate information about the different feeding options among others. Our study noted that mothers who were knowledgeable about the prevention of mother-to-child transmission were 16 times more likely to practice recommended infant feeding compared to mothers who did not have the knowledge. Mothers knowledgeable about PMTCT had the backup reasons as to breastfeed exclusively compared to their counterparts. This finding can be related to a study done in Ethiopia which showed that mothers with sufficient knowledge about PMTCT were more likely to practice EBF [21], however on the contrary another study showed knowledge on PMTCT were not associated with EBF [20].

The strengths of this study included using random sampling method that enabled generalisation of results since the study population was highly representative. The study used both qualitative and quantitative data, we pretested the questionnaire hence doing corrections where necessary, it had a very high response rate, in data analysis logistical regression was done. We also worked with an independent statistician who was not part of the study design, data collection. We blinded this statistician by coding all the data that was collected and this helped to avoid any biases that would emerge. However it had the following limitations; the sample size was small, It was an undergraduate project with minimal funding thereby affecting sample size and the scope of the study; Data were collected from Only one HCIV and so the results may not represent entire Gulu or Uganda. The study was hospital-based and the results are only limited to those who came to Awach not entire sub county and possibility of social desirability bias. Only two FGD's were done limiting on the data gathered. The data collection tools were not standard tools by either Ministry of Health Uganda or world health organisation and therefore our results may not have external validity.

## Conclusions and recommendations

Majority of HIV-positive mothers in Awach practice the recommended infant feeding practices. However, there is still a knowledge gap on whether HIV is transmitted through pregnancy, delivery or breast feeding, the factors associated with the choice of infant feeding practice in Awach included education level, place of delivery and knowledge on PMTCT. We therefore recommend the Ministry of Health to increase funding on PMTCT services. We also recommend the health facility to Hold sensitisations of the mothers by mid wives at least weekly. We highly recommend the mothers to deliver from health facilities, since mothers who delivered from health facilities were more likely to practice the recommended breastfeeding practices than their counter parts. The Mothers should follow the recommended guidelines and should attend ANC. More research about this topic involving a large sample size and different hospitals should be done to bring out a clear picture.

## Data Availability

All the project materials and data about this project are available. These can be accessed by contacting the first author (Ivan Mutawulira) on ivomutawulira@gmail.com.

## Supplementary material

For supplementary material accompanying this paper visit http://doi.org/10.1017/S0950268822001091. click here to view supplementary material

## Declarations

We declare that this report entitled ‘infant feeding practices and associated factors among HIV positive mothers attending EID clinic from Awach Health Centre IV, Gulu Uganda’ was our work composed solely by us, undersigned and to the best of our knowledge has never been submitted to any institution of higher learning for any award and where it was stated we referenced and cited it. Abel Wilson Walekhwa is an associate Editor for Epidemiology and Infection journal, University of Cambridge Press. Therefore, the work presented is entirely our own.
