# Supplementary material for: Exploring infant feeding practices and associated factors among HIV-positive mothers attending early infant diagnosis clinic in Northern Uganda
Source: Epidemiol Infect. 2022 Jun 20;150:e130. doi: 10.1017/S0950268822001091 (PMC9306008; doi:10.1017/S0950268822001091)
Supplement: Supplementary file 1 [file S0950268822001091sup001.pdf]

GULU UNIVERSITY

P.O. Box 166, Gulu (U)
Website: www.gu.ac.ug
Email: gurec@gu.ac.ug

Tel: +256 471 432 096
Fax: +256 471 432 913
Mob: +256 772 305 621
+256 776 812 147

RESEARCH ETHICS COMMITTEE

14/10/2021

To: Muharabu Laymond

Gulu University
0784619844

Type: Initial Review

Re: GUREC-2021-76: Infant feeding practices and associated factors among HIV positive mothers attending ART clinic from Awach HC III,in Gulu Uganda. , 3.0, 2021-10-14

I am pleased to inform you that at the **76th** convened meeting on **19/08/2021**, the Gulu University REC, committee meeting, etc voted to approve the above referenced application.
Approval of the research is for the period of **14/10/2021** to **14/10/2022.**

As Principal Investigator of the research, you are responsible for fulfilling the following requirements of approval:

1. All co-investigators must be kept informed of the status of the research.
2. Changes, amendments, and addenda to the protocol or the consent form must be submitted to the REC for re-review and approval **prior** to the activation of the changes.
3. Reports of unanticipated problems involving risks to participants or any new information which could change the risk benefit: ratio must be submitted to the REC.
4. Only approved consent forms are to be used in the enrollment of participants. All consent forms signed by participants and/or witnesses should be retained on file. The REC may conduct audits of all study records, and consent documentation may be part of such audits.
5. Continuing review application must be submitted to the REC **eight weeks** prior to the expiration date of **14/10/2022** in order to continue the study beyond the approved period. Failure to submit a continuing review application in a timely fashion may result in suspension or termination of the study.
6. The REC application number assigned to the research should be cited in any correspondence with the REC of record.
7. You are required to register the research protocol with the Uganda National Council for Science and Technology (UNCST) for final clearance to undertake the study in Uganda.

The following is the list of all documents approved in this application by Gulu University REC:

No.	Document Title	Language	Version Number	Version Date
1	COVID-19 Risk Management Plan	English	3.0	2021-10-14
2	Data collection tools	English	3.0	2021-10-14
3	Data collection tools	English	3.0	2021-10-14
4	Data collection tools	English	3.0	2021-10-14
5	Informed Consent forms	Acholi	3.0	2021-10-14
6	Informed Consent forms	English	3.0	2021-10-14
7	Protocol	English	3.0	2021-10-14

Yours Sincerely

Julaina A. Obika (PhD)
For: Gulu University REC
